# Unmet need for family planning and associated factors among currently married women in Nepal: A further analysis of Nepal Demographic and Health Survey—2022

**DOI:** 10.1371/journal.pone.0303634

**Published:** 2024-05-31

**Authors:** Saugat Pratap K. C., Bikram Adhikari, Achyut Raj Pandey, Merina Pandey, Sampurna Kakchapati, Santosh Giri, Shreeman Sharma, Bipul Lamichhane, Ghanshyam Gautam, Deepak Joshi, Bishnu Prasad Dulal, Shophika Regmi, Sushil Chandra Baral

**Affiliations:** Research, Evaluation and Innovation Department, HERD International, Kathmandu, Nepal; Tropical Health and Education Trust, UK/Nepal based, NEPAL

## Abstract

**Introduction:**

Family planning (FP) is crucial for improving maternal and newborn health outcomes, promoting gender equality, and reducing poverty. Unmet FP needs persist globally, especially in South Asia and Sub-Saharan Africa leading to unintended pregnancies, unsafe abortions, and maternal fatalities. This study aims to identify the determinants of unmet needs for FP from a nationally representative survey.

**Methods:**

We analyzed the data of 11,180 currently married women from nationally representative Nepal Health Demographic Survey 2022. We conducted weighted analysis in R statistical software to account complex survey design and non-response rate. We conducted univariate and multivariable binary and multinomial logistic regression to assess association of unmet need for FP with independent variables including place of residence, province, ecological belt, ethnicity, religion, current age, participant’s and husband’s education, occupation, wealth quintile, parity, desire for child, and media exposure.

**Results:**

The total unmet FP need was 20.8% (95%CI: 19.7, 21.9) accounting 13.4% (95%CI: 12.5, 14.4) for unmet need for limiting and 7.4% (95%CI: 6.8, 8.0) for unmet for spacing. Lower odds of total unmet need for FP were present in 20–34 years and 35–49 years compared to <20 years, women belonging to Madhesi ethnic group (AOR: 0.78; 95%CI: 0.64, 0.95) compared to Brahmin/Chhetri, women from richest (AOR: 0.69; 95%CI: 0.56, 0.84), richer (AOR: 0.82; 95%CI: 0.68, 0.97) and middle wealth quintile (AOR: 0.82; 95%CI:0.70, 0.98) groups compared poorest wealth quintile group and women belonging to rural area (AOR: 0.89; 95%CI: 0.80, 0.99) compared to urban area. Higher odds of unmet need for FP were present among women with basic (AOR: 1.34; 95%CI: 1.17, 1.54), and secondary level (AOR: 1.32; 95%CI: 1.12, 1.56) education compared to women without education, among women from Madhesh (AOR: 1.56; 95%CI: 1.22, 1.98), Gandaki (AOR: 2.11; 95%CI: 1.66, 2.68), Lumbini (AOR: 1.97; 95%CI: 1.61, 2.42) and Sudurpashchim province (AOR: 1.64; 95%CI: 1.27, 2.10) compared to Koshi province and among women whose husband education was basic level *(AOR*:1.37; 95%CI: 1.15, 1.63), or secondary level *(AOR*: 1.32; 95%CI: 1.09, 1.60) education.

**Conclusion:**

Nepal faces relatively high unmet FP needs across various socio-demographic strata. Addressing these needs requires targeted interventions focusing on age, ethnicity, religion, education, and socio-economic factors to ensure universal access to FP services.

## Introduction

Family planning (FP) is a crucial aspect of reproductive health, enabling individuals to make informed choices about when to have children and how many to have [1]. Ensuring universal access to FP is a fundamental human right that plays a central role in promoting gender equality and empowering women. Additionally, it serves as a crucial factor in alleviating poverty and advancing the objective of achieving Universal Health Coverage (UHC) [2]. The World Health Organization (WHO) defines unmet FP needs as fecund and sexually active women who desire to postpone or limit childbearing but are not using any contraceptive method [3]. In 2019, despite the various advantages of FP and efforts to enhance accessibility, approximately 160 million women and adolescents worldwide still lacked access to adequate family planning services. More than half of these women with unmet needs resided in Sub-Saharan Africa and South Asia [4].

The presence of a significant unmet need for FP results in elevated rates of unintended pregnancies, which, in turn, are closely linked to unsafe abortions and maternal fatalities. These connections are well-established in the realm of public health research [5, 6]. Addressing the persistent demand for FP has remained a core focus of global health and population strategies for many years [7]. Empowering women to make their own decisions regarding pregnancy, rather than being forced upon, can lead to broader social and economic advantages extending beyond the health sector. These benefits include higher levels of education, increased participation of women in the workforce, and greater accumulation of wealth within households [8]. Addressing the unmet need for FP aligns with two Sustainable Development Goals (SDGs). SDG 3 focuses on promoting well-being and ensuring universal access to sexual and reproductive health-care services, including the integration of reproductive health into national strategies and programs. The SDG 5 aims to achieve gender equality and empower women and girls by guaranteeing universal access to sexual and reproductive health services and reproductive rights [9].

Multiple factors have been identified in the literature as crucial elements associated with unmet need for FP among women in developing countries [10]. In Nepal, multiple studies based on the Demographic and Health Survey 2011 have revealed a high prevalence of unmet need for family planning [11, 12], with disparities based on education and age [11]. Additionally, research conducted in Nepal has shown that the proportion of contraceptive users decreases with longer travel times to access family planning outlets, impacting both urban and rural women [13, 14]. Women’s societal position and decision-making power are closely intertwined with family planning access and utilization. Despite efforts by leading health organizations to address inequities in unmet need for family planning, the problem persists [15], indicating the need for further action. Thus, this study assesses the prevalence of unmet need for family planning for limiting and spacing births and identify the socio-demographic factors associated with it.

## Methods

### Study design

We analyzed Nepal Demographic and Health Survey, 2022 (NDHS 2022) dataset of currently married women (who have been married and are not either divorced, widowed or separated) in this study. NDHS 2022 is a nationally representative survey implemented by New ERA under the aegis of the Ministry of Health and Population (MoHP) with the technical support of ICF International. NDHS 2022 was funded by the US Agency for International Development (USAID).

### Study setting

Nepal, positioned in Southeast Asia, is a landlocked country spanning an area of 147,516 km2. It is divided into seven administrative provinces, encompassing a total of 753 municipalities, including 6 metropolitan cities, 11 sub-metropolitan cities, 276 urban municipalities, and 460 rural municipalities. The geographical landscape of Nepal consists of three ecological regions: Mountain, Hill, and Terai. According to the National Population and Housing Census of 2021, Nepal’s total population reached 29,164,578 individuals, with females accounting for 14,911,027 (51.1%). The Human Development Index of rural and urban Nepal were 0.647 and 0.561 respectively with overall human development index of 0.587.

### Sample and sampling

The sampling and sampling technique of NDHS 2022 is described elsewhere [16]. The samples of NDHS are nationally representative, encompassing all seven provinces of the nation. The initial stratification involved dividing each province into urban and rural areas, creating a sampling stratum for each province. This led to the establishment of 14 sampling strata in total. The sampling process comprised two stages. Initially, 476 primary sampling units (PSUs) were chosen using a probability-proportional-to-size approach. Out of these, 248 PSUs were from urban regions, and 228 were from rural regions. Subsequently, 30 households were selected from each PSU in the second stage, resulting in an overall sample size of 14,280 households, with 7,440 from urban areas and 6,840 from rural areas. Among the households surveyed, there were 15,283 eligible women aged 15–49 for individual interviews. Interviews were successfully conducted with 14,845 of these women, yielding a response rate of 97%. For the purposes of this study, data from 11,180 currently married women were included.

### Data collection

Data collection for NDHS 2022 was done by 19 teams between January 5 and June 22, 2022. Each team was comprised of a supervisor, a male interviewer, three female interviewers, and a biomarker specialist.

#### Dependent variables

*Unmet need for FP*. The adolescents women who were not pregnant and not postpartum amenorrheic and are considered fecund and want to postpone their next birth for 2 or more years or stop childbearing altogether but are not using a contraceptive method, or have a mistimed or unwanted current pregnancy, or are postpartum amenorrheic and their most recent birth in the last 2 years was mistimed or unwanted were considered to have unmet need for family planning [16]. Unmet need of family planning is categorized into three categories- unmet need for spacing (women who wanted to wait or delay in having another child but not using any form of contraception), unmet need for limiting (women who did not want any more children but not using any form of contraception), and no unmet need. Unmet need for family planning was recategorized into two categories- met need for family planning and total unmet need for family planning by merging two categories (unmet need for limiting and spacing).

#### Independent variables

The independent variables assessed in this study included ecological belt (Mountain/Hill/Terai), setting (Urban/Rural), province(Koshi/Madhesh/Bagmati/Gandaki/Lumbini/Karnali/Sudurpashchim), age (in years), ethnicity (Brahmin or Chhetri/ Dalit/Janajatis/Madhesi/Muslim and others) as the NDHS classifies the ethnicity into groups such as Brahmin/Chhetri, Janajatis, Madhesis, and Muslims to understand and address the diverse social and cultural needs of the population, religion (Hindu/Non-Hindu), wealth quintile (Poorest/Poorer/Middle/Richer/Richest), education(No education/Basic/Secondary/Higher), occupation (Not working/Agriculture/Professional or technical or manager or clerical), and health insurance(Covered/Not covered), media exposure [this was taken as health messages are often communicated via media](Present/Not present), husband/partner’s education level (No education, basic, secondary, higher, don’t know).

#### Statistical analysis

We performed pre-analytical and statistical analysis using R version 4.2.0. We performed a weighted analysis was used using “survey package” [17] to account complex survey design of NDHS 2022. We presented categorical variables as weighted frequency, weighted percent and their 95% confidence interval (CI) whereas numerical variables as mean and 95% CI. We used univariable and multivariable, binary, and multinomial logistic regression to determine the association of unmet need of FP with independent variables. We used “multinom” function from “nnet” package [18] accounting weight to perform univariable and multivariable multinomial logistic regression. The results of the regression analysis were presented as crude odds ratio (COR), adjusted odds ratio (AOR), and 95% CI.

#### Ethical approval

We requested the DHS program for permission to use NDHS 2022 dataset [18] which was granted to download and use NDHS 2022 dataset from https://www.dhsprogram.com. NDHS 2022 obtained ethical approval from the institutional review board of ICF International, United States of America (Reference number: 180657.0.001.NP.DHS.01, Date: 28^th^ April 2022) and the ethical review board of Nepal Health Research Council (Reference number: 678, Date: 30^th^ September 2021). Our analysis is based on publicly available dataset of NDHS 2022. In the survey, written informed consent was obtained from all adult participants and consent/assent was obtained from parents and guardians for participants below age of 18 years.

## Results

Table 1 shows the socio-economic characteristics of married women. More than two-third of women (67.6%) resided in urban areas. More than half of the women (56.1%) were from the terai region. Madhesh province had the highest representation at 21.6%, followed by Bagmati (19.3%), Lumbini (18.3%) and Koshi province (16.9%). Slightly more than one-third (36.2%) of the women belonged to Janajati followed by Brahmin/Chettri (27.1%), Madheshi (16.4%), and Dalit (15.5%). Majority (83.5%) of the women were Hindu. The median age of the women is 32.0 years and most of the women (53.7%) belonged to 20–34 years age group. The distribution of married women across wealth quintiles revealed that a significant portion, 21.3% belonged to the richer quintile, followed closely by the middle (20.8%), richest (19.9%), poorer (19.8%), and poorest quintiles (18.2%). About 38% of the women’s husbands had secondary education, while 13.3% were uneducated. According to women’s occupation, more than half (53%) of the surveyed women were engaged in agriculture and 23.9% of women were not working. 49.8% of the women had exposure to media. The majority of women (72.5%) did not desire more children. Most women (66.5%) were multipara, primipara and nullipara women constituted 24.1% and 9.4% respectively.

**Table 1:** Characteristics of currently married women.

Characteristic	n	% (95%CI)
**Place of residence**		
Urban	7,553	67.6 (66.1, 69.0)
Rural	3,627	32.4 (31.0, 33.9)
**Ecological region**		
Mountain	629	5.6 (3.92, 8.01)
Hill	4,275	38.2 (34.5, 42.1)
Terai	6,276	56.1 (52.4, 59.8)
**Province**		
Koshi	1,887	16.9 (15.7, 18.2)
Madhesh	2,419	21.6 (20.4, 23.0)
Bagmati	2,156	19.3 (17.7, 21.0)
Gandaki	1,046	9.4 (8.32, 10.5)
Lumbini	2,020	18.1 (16.9, 19.3)
Karnali	691	6.2 (5.66, 6.75)
Sudurpashchim	960	8.6 (7.93, 9.30)
**Ethnicity**		
Brahmin/Chhetri	3,031	27.1 (24.9, 29.5)
Dalit	1,734	15.5 (13.7, 17.5)
Janajati	4,042	36.2 (33.4, 39.0)
Madheshi	1,835	16.4 (14.3, 18.7)
Muslim & others	539	4.8 (3.42, 6.74)
**Religion**		
Hindu	9,338	83.5 (81.4, 85.4)
Non-Hindu	1,842	16.5 (14.6, 18.6)
**Current age (in years)**		
<20 years	563	5 (4.52, 5.60)
20–34 years	6,007	53.7 (52.6, 54.9)
35–49 years	4,609	41.2 (40.1, 42.4)
**Wealth index**		
Poorest	2,031	18.2 (16.3, 20.2)
Poorer	2,217	19.8 (18.1, 21.7)
Middle	2,323	20.8 (19.3, 22.4)
Richer	2,381	21.3 (19.7, 23.0)
Richest	2,228	19.9 (17.6, 22.5)
**Highest educational level**		
No education	3,475	31.1 (29.4, 32.9)
Basic	3,701	33.1 (31.8, 34.5)
Secondary	3,536	31.6 (30.1, 33.2)
Higher	468	4.2 (3.59, 4.88)
**Husband/partner’s education level**		
No education	1,482	13.3 (12.1, 14.5)
Basic	4,470	40 (38.3, 41.6)
Secondary	4,250	38 (36.5, 39.6)
Higher	822	7.3 (6.45, 8.36)
Don’t know	156	1.4 (1.11, 1.75)
**Occupation**		
Not working	2,677	23.9 (22.1, 25.9)
Agriculture	5,920	53 (50.1, 55.8)
Professional, technical, managerial or clerical	734	6.6 (5.83, 7.38)
Sales and service	928	8.3 (7.43, 9.26)
Skilled/unskilled labor	907	8.1 (7.24, 9.08)
Other	14	0.1 (0.05, 0.28)
**Media exposure**	5,568	49.8 (47.8, 51.8)
**Desire for child**		
Having another	2,681	24 (22.9, 25.1)
Undecided	394	3.5 (3.07, 4.05)
Wants no more	8,104	72.5 (71.3, 73.7)
**Parity**		
Multipara	7,429	66.5 (65.3, 67.6)
Primipara	2,696	24.1 (23.1, 25.1)
Nullipara	1,055	9.4 (8.7, 10.2)

%: weighted percentage; n: weighted frequency; CI: confidence interval

Table 2 presents unmet need of FP among different subcategories. The unmet need for spacing births was higher in the urban areas (7.5%) than in the rural area (7.2%), while the unmet need for limiting births is higher in rural areas (13.9%) compared to urban areas (13.2%). The hill region had the highest unmet need for limiting births (16.1%), while the mountain region had the highest unmet need for spacing births (7.9%). The Gandaki province stood out with the highest total unmet need (28.1%), as well as the highest unmet need for limiting births (20.6%). On the other hand, the Bagmati province had the lowest total unmet need (16%) and the lowest unmet need for spacing births (4.3%). The Dalit ethnic group had the highest total unmet need (25.5%), with the majority of it being unmet need for spacing births (11%). Unmet need for FP is highest among women aged below 20 years (30.9%) and decreases with age. The majority of their unmet need is for spacing births (28.4%). The poorest women had the highest total unmet need (24.7%), while the richest women had the lowest total unmet need (16.9%). Women with no education had the lowest unmet need for spacing births (3.2%) and the highest unmet need for limiting births (13.2%). The unmet need for limiting births was the highest among agricultural workers (14.7%) and the lowest among those in sales and service occupations (11.1%).

**Table 2:** Unmet need of family planning among different subcategories.

Characteristic	n	Total unmet need % (95%CI)	Unmet need for spacing, % (95%CI)	Unmet need for limiting, % (95%CI)
**Place of residence**				
Urban	7,553	20.7 (19.2, 22.1)	7.5 (6.66, 8.39)	13.2 (12.0, 14.4)
Rural	3,627	21.1 (19.6, 22.7)	7.2 (6.41, 7.97)	13.9 (12.7, 15.3)
**Ecological region**				
Mountain	629	19.1 (16.1, 22.5)	7.9 (6.15, 10.2)	11.2 (8.99, 13.8)
Hill	4,275	22.7 (21.2, 24.4)	6.6 (5.85, 7.44)	16.1 (14.7, 17.6)
Terai	6,276	19.7 (18.1, 21.3)	7.8 (6.91, 8.89)	11.8 (10.6, 13.1)
**Province**				
Koshi	1,887	17.6 (15.7, 19.6)	7.9 (6.48, 9.64)	9.6 (8.29, 11.2)
Madhesh	2,419	21.1 (18.4, 24.1)	9.9 (8.17, 12.0)	11.2 (9.30, 13.4)
Bagmati	2,156	16 (14.0, 18.2)	4.3 (3.36, 5.38)	11.7 (9.89, 13.9)
Gandaki	1,046	28.1 (24.8, 31.5)	7.4 (5.94, 9.29)	20.6 (17.7, 23.9)
Lumbini	2,020	23.3 (20.3, 26.6)	6.8 (5.56, 8.39)	16.5 (14.0, 19.4)
Karnali	691	23.4 (20.8, 26.2)	8.5 (6.89, 10.4)	14.9 (12.8, 17.3)
Sudurpashchim	960	22.1 (19.1, 25.3)	7.2 (5.99, 8.62)	14.9 (12.4, 17.8)
**Ethnicity**				
Brahmin/Chhetri	3,031	20.7 (18.9, 22.7)	6.2 (5.33, 7.16)	14.6 (13.1, 16.2)
Dalit	1,734	25.5 (23.3, 28.0)	11 (9.45, 12.8)	14.5 (12.5, 16.8)
Janajati	4,042	19.7 (18.1, 21.5)	6.5 (5.66, 7.54)	13.2 (11.9, 14.7)
Madheshi	1,835	17.6 (14.9, 20.5)	6.7 (5.37, 8.22)	10.9 (8.76, 13.5)
Muslim & others	539	24.8 (19.3, 31.2)	11.1 (7.22, 16.7)	13.7 (10.4, 17.8)
**Religion**				
Hindu	9,338	20.4 (19.3, 21.6)	7.1 (6.45, 7.77)	13.3 (12.4, 14.3)
Non-Hindu	1,842	22.8 (20.2, 25.6)	8.8 (7.05, 11.0)	13.9 (12.0, 16.1)
**Current age (in years)**				
<20 years	563	30.9 (26.8, 35.4)	28.4 (24.3, 32.8)	2.6 (1.62, 4.02)
20–34 years	6,007	24.7 (23.3, 26.1)	10.8 (9.89, 11.8)	13.9 (12.8, 15.0)
35–49 years	4,609	14.5 (13.2, 15.9)	0.3 (0.17, 0.66)	14.2 (12.9, 15.5)
**Wealth index**				
Poorest	2,031	24.7 (22.7, 26.8)	8.7 (7.48, 10.0)	16.1 (14.4, 17.8)
Poorer	2,217	21.4 (19.3, 23.6)	8.6 (7.40, 10.0)	12.7 (11.1, 14.6)
Middle	2,323	20.4 (18.5, 22.6)	7.3 (6.19, 8.52)	13.2 (11.6, 15.0)
Richer	2,381	20.9 (18.9, 23.1)	7.7 (6.28, 9.37)	13.3 (11.6, 15.1)
Richest	2,228	16.9 (14.5, 19.6)	4.7 (3.81, 5.86)	12.2 (10.0, 14.7)
**Highest educational level**				
No education	3,475	16.4 (14.7, 18.2)	3.2 (2.34, 4.34)	13.2 (11.7, 14.8)
Basic	3,701	23.7 (22.1, 25.4)	8.1 (7.12, 9.15)	15.6 (14.3, 17.1)
Secondary	3,536	22.7 (21.0, 24.6)	11 (9.81, 12.4)	11.7 (10.5, 13.1)
Higher	468	16.1 (12.0, 21.2)	5.3 (3.12, 8.75)	10.8 (7.55, 15.2)
**Husband/partner’s education level**				
No education	1,482	15.3 (13.0, 18.0)	4.4 (3.22, 5.98)	10.9 (9.02, 13.1)
Basic	4,470	22.2 (20.7, 23.8)	7 (6.10, 8.00)	15.2 (14.0, 16.5)
Secondary	4,250	21.8 (20.3, 23.5)	8.9 (7.88, 9.94)	13 (11.7, 14.4)
Higher	822	16.6 (13.8, 19.8)	5.5 (4.04, 7.43)	11.1 (8.88, 13.8)
Don’t know	156	26.1 (18.3, 35.8)	16 (10.4, 23.9)	10.1 (6.12, 16.1)
**Parity**				
Multipara	7,429	19.7 (18.4, 21.1)	2.8 (2.28, 3.32)	17 (15.7, 18.3)
Primipara	1,055	16 (13.6, 18.8)	15.6 (13.2, 18.3)	0.5 (0.17, 1.23)
Nullipara	2,696	25.7 (23.8, 27.7)	16.9 (15.2, 18.7)	8.8 (7.63, 10.1)
**Total**	11,180	20.8 (19.7, 21.9)	13.4 (12.5, 14.4)	7.4 (6.8, 8.0)

%: weighted percentage; n: weighted frequency; CI: confidence interval

Table 3 highlights the further statistical analysis of the data which reveals that the women belonging to 20–34 years and 35–49 years age group had lower odds of total unmet need for FP and unmet need spacing compared to <20 years age group. Women belonging to Madheshi ethnicity had 22% (AOR: 0.78;95%CI: 0.64, 0.95) lower odds of total unmet need for FP and 30% (AOR: 0.70; 95%CI: 0.51, 0.96) lower odds of unmet need for spacing compared to Brahmin/Chettri. Women who were non-Hindus had 21% higher odds to have unmet need for limiting (AOR: 1.21; 95%CI: 1.00, 1.47) and 37% higher odds of unmet need for spacing (AOR: 1.37; 95%CI: 1.05, 1.78) compared to Hindu women. The odds of total unmet need for family planning were 1.34 (95%CI: 1.17, 1.54) times among women with basic level education and 1.32 (95%C: 1.12, 1.56) times among women with secondary level education compared to women without formal education. Similarly, the odds of unmet need for spacing was higher among women who attained basic education (AOR:1.88; 95%CI: 1.44, 2.45), secondary education (AOR: 2.72; 95%CI: 2.03, 3.65) and higher education (AOR: 2.32; 95%CI: 1.35, 4.00) compared with their counterparts. Women belonging to richest wealth quintile households were less likely to have total unmet need for FP, unmet need for spacing and limiting. Women from the hill region were 1.24 times likely to have total unmet need (AOR:1.24; 95%I: 1.01, 1.54) and 1.44 times likely to have unmet need for limiting (AOR: 1.44; 95%CI: 1.09, 1.89). Women residing in Madhesh (AOR: 1.56; 95%CI: 1.22, 1.98)), Gandaki (AOR: 2.11; 95%CI: 1.66, 2.68), Lumbini (AOR: 1.97; 95%CI: 1.61, 2.42) and Sudurpashchim province (AOR: 1.64; 96%CI: 1.27, 2.10) had higher odds of unmet need for limiting compared to Koshi province. Women from Madhesh province have 1.71 (95%CI: 1.29, 2.28) times more likely and women from Bagmati have 41% less likely to have (AOR:0.59; 95%CI: 0.43, 0.80) to have unmet need for spacing.

**Table 3:** Factors associated with unmet need for FP.

	Total unmet need ^&^		Total met need vs Unmet need for limiting #		Total met need vs Unmet need for spacing #	
	COR (95%CI)	AOR (95%CI)	COR (95%CI)	AOR (95%CI)	COR (95%CI)	AOR (95%CI)
***Age***			0			
<20 years	Ref	Ref	Ref	Ref	Ref	Ref
20–34 years	0.73 (0.61, 0.89)*	0.83 (0.69, 1.01)	4.97 (2.92, 8.46)**	5.46 (3.20, 9.31)**	0.35 (0.29, 0.43)**	0.43 (0.35, 0.54)**
35–49 years	0.38 (0.31, 0.46)**	0.49 (0.40, 0.61)**	4.47 (2.63, 7.62)**	5.15 (3.00, 8.84)**	0.01 (0.01, 0.02)**	0.02 (0.01, 0.03)**
***Ethnicity***						
Brahmin/Chhetri	Ref	Ref	Ref	Ref	Ref	Ref
Dalit	1.31 (1.14, 1.51)**	1.13 (0.96, 1.32)	1.06 (0.90, 1.26)	0.99 (0.81, 1.19)	1.89 (1.53, 2.34)**	1.48 (1.14, 1.91)*
Janajati	0.94 (0.84, 1.06)	0.88 (0.77, 1.01)	0.9 (0.78, 1.03)	0.85 (0.73, 1.00)*	1.04 (0.86, 1.27)	0.96 (0.76, 1.21)
Madheshi	0.81 (0.70, 0.94)*	0.78 (0.64, 0.95)*	0.72 (0.60, 0.86)**	0.86 (0.68, 1.08)	1.04 (0.82, 1.31)	0.70 (0.51, 0.96)*
Muslim & Others	1.26 (1.01, 1.56)*	1.07 (0.79, 1.46)	0.99 (0.76, 1.29)	1.07 (0.74, 1.55)	1.89 (1.39, 2.58)**	1.21 (0.75, 1.96)
***Religion***						
Hindu	Ref	Ref	Ref	Ref	Ref	Ref
Non-Hindu	1.15 (1.02, 1.30)*	1.26 (1.07, 1.48)*	1.08 (0.93, 1.25)	1.21 (1.00, 1.47)*	1.29 (1.07, 1.54)*	1.37 (1.05, 1.78)*
***Highest educational level***						
No education	Ref	Ref	Ref	Ref	Ref	Ref
Basic	1.59 (1.41, 1.78)**	1.34 (1.17, 1.54)**	1.30 (1.14, 1.48)**	1.25 (1.07, 1.46)*	2.77 (2.22, 3.47)**	1.88 (1.44, 2.45)**
Secondary	1.50 (1.33, 1.69)**	1.32 (1.12, 1.56)**	0.96 (0.83, 1.11)	0.97 (0.80, 1.18)	3.73 (3.01, 4.64)**	2.72 (2.03, 3.65)**
Higher	0.98 (0.75, 1.26)	1.14 (0.83, 1.57)	0.82 (0.60, 1.11)	0.88 (0.60, 1.29)	1.64 (1.05, 2.57)*	2.32 (1.35, 4.00)*
***Wealth quintile***						
Poorest	Ref	Ref	Ref	Ref	Ref	Ref
Poorer	0.83 (0.72, 0.95)*	0.87 (0.74, 1.02)	0.76 (0.64, 0.90)*	0.83 (0.68, 1.00)	0.95 (0.77, 1.18)	0.93 (0.73, 1.19)
Middle	0.78 (0.68, 0.90)**	0.82 (0.70, 0.98)*	0.78 (0.65, 0.92)*	0.85 (0.69, 1.03)	0.79 (0.64, 0.99)*	0.76 (0.58, 1.00)
Richer	0.81 (0.70, 0.93)*	0.82 (0.68, 0.97)*	0.79 (0.66, 0.93)*	0.84 (0.68, 1.03)	0.84 (0.68, 1.05)	0.75 (0.57, 1.00)
Richest	0.62 (0.53, 0.72)*	0.69 (0.56, 0.84)**	0.69 (0.58, 0.82)**	0.73 (0.58, 0.93)*	0.49 (0.38, 0.64)**	0.60 (0.43, 0.85)*
***Place of residence***						
Urban	Ref	Ref	Ref	Ref	Ref	Ref
Rural	1.03 (0.93, 1.13)	0.89 (0.80, 0.99)*	1.06 (0.95, 1.20)	0.9 (0.79, 1.03)	0.96 (0.82, 1.12)	0.85 (0.71, 1.01)
***Ecological region***						
Mountain	Ref	Ref	Ref	Ref	Ref	Ref
Hill	1.24 (1.01, 1.54)*	1.22 (0.98, 1.53)	1.5 (1.16, 1.95)*	1.44 (1.09, 1.89)*	0.87 (0.64, 1.20)	0.9 (0.64, 1.27)
Terai	1.04 (0.84, 1.28)	0.91 (0.71, 1.18)	1.06 (0.82, 1.38)	0.99 (0.73, 1.35)	1 (0.73, 1.35)	0.8 (0.54, 1.18)
***Province***						
Koshi	Ref	Ref	Ref	Ref	Ref	Ref
Madhesh	1.26 (1.08, 1.47)*	1.62 (1.34, 1.95)**	1.21 (0.99, 1.48)	1.56 (1.22, 1.98)**	1.31 (1.05, 1.62)*	1.71 (1.29, 2.28) **
Bagmati	0.89 (0.76, 1.05)	0.87 (0.72, 1.06)	1.19 (0.98, 1.46)	1.08 (0.86, 1.36)	0.53 (0.40, 0.69)**	0.59 (0.43, 0.80) **
Gandaki	1.83 (1.53, 2.19)**	1.65 (1.35, 2.02)**	2.45 (1.97, 3.04)**	2.11 (1.66, 2.68)**	1.08 (0.81, 1.44)	1.02 (0.73, 1.43)
Lumbini	1.43 (1.22, 1.67)**	1.58 (1.33, 1.86)**	1.84 (1.51, 2.23)**	1.97 (1.61, 2.42)**	0.93 (0.73, 1.18)	1.04 (0.80, 1.35)
Karnali	1.43 (1.16, 1.77)**	1.11 (0.88, 1.41)	1.66 (1.28, 2.16)**	1.33 (0.99, 1.77)	1.15 (0.84, 1.58)	0.82 (0.57, 1.20)
Sudurpashchim	1.33 (1.10, 1.61)*	1.36 (1.10, 1.67)*	1.63 (1.29, 2.07)**	1.64 (1.27, 2.10)**	0.96 (0.71, 1.30)	0.96 (0.69, 1.33)
***Husband/partner’s education level***						
No education	Ref	Ref	Ref	Ref	Ref	Ref
Basic	1.58 (1.35, 1.85**)	1.37 (1.15, 1.63)**	1.52 (1.26, 1.82)**	1.46 (1.20, 1.78)**	1.73 (1.32, 2.28)**	1.07 (0.78, 1.46)
Secondary	1.55 (1.32, 1.82)**	1.32 (1.09, 1.60)*	1.29 (1.07, 1.55)*	1.34 (1.08, 1.68)*	2.18 (1.66, 2.86)**	1.15 (0.83, 1.61)
Higher	1.1 (0.87, 1.39)	1.15 (0.87, 1.52)	1.04 (0.79, 1.36)	1.25 (0.90, 1.73)	1.27 (0.86, 1.88)	0.92 (0.57, 1.47)
Don’t know	1.95 (1.32, 2.84)*	1.55 (1.05, 2.30)*	1.06 (0.61, 1.83)	1.08 (0.62, 1.88)	4.18 (2.54, 6.88)**	2.04 (1.19, 3.49)*
***Media Exposure***						
No	Ref	Ref	Ref	Ref	Ref	Ref
Yes	0.91 (0.83, 1.00)*	0.93 (0.84, 1.02)	0.93 (0.83, 1.04)	0.93 (0.82, 1.04)	0.88 (0.76, 1.01)	0.93 (0.80, 1.09)

COR: Crude Odds ratio; AOR: Adjusted odds ratio; CI: Confidence interval; Ref: reference group

* Significance at 0.05 level of significance

** significance at 0.001 level of significance

&: Binary logistic regression

#: Multinomial logistic regression

The total unmet need for family planning is higher among women whose husband education was basic level (AOR:1.37; 95%CI: 1.15, 1.63)), or secondary level (AOR: 1.32; 95%CI: 1.09, 1.60) education compared to women whose husband has no formal education. Similarly, the odds of unmet need for limiting were higher in women whose husband has basic level (AOR: 1.46; 95%CI: 1.20, 1.78) and secondary level (AOR: 1.34; 95%CI: 1.08, 1.68) education compared to women whose husband no formal education. Our multivariable logistic regression results highlight significant associations between socio-demographic factors and unmet family planning needs.

## Discussion

The overall aim of this study was to identify associated factors determining unmet need for FP among currently married women in Nepal. The total unmet FP need was 20.8%, unmet need for limiting was 13.4% and unmet need for spacing was 7.4%. We found association of unmet need of FP with age, ethnicity, religion, participant’s and partner’s education level, province, occupation, wealth quintile, parity and desire for child.

Among South Asian countries, the unmet need of FP in Nepal, which is reported to be 20.8% in our study, is higher than Bangladesh (12% in 2017–18), India (9.4% in 2019–21) and Pakistan (17.3% in 2017–19) [19]. This variance might plausibly stem from differences in the accessibility of health services, awareness, and attitudes towards FP provisions, coupled with influences from socioeconomic, demographic, and cultural aspects. The unmet need of FP in Nepal in 2016 was 24% [20], which is higher than our study, which could possibly be due to government of Nepal’s commitment to uphold and continue all efforts launched through the implementation of the FP2020 commitments by focusing on reaching the unreached.

The current study demonstrates that women below the age of 20 exhibit higher unmet need for FP in total and unmet need for spacing compared to their counterparts. These findings resonate with other studies in India and Ethiopia [21–23]. This pattern can potentially be attributed to the relative immaturity of women in this age bracket, leading to challenges in decision-making regarding FP, alongside potential difficulties in overcoming the influences from spouses, in-laws, and the broader community.

However, the proportion of unmet need for limiting is higher among women aged 20 to 39 years. Relatively higher unmet need for limiting among 20–34 years seem reasonable, as they may have already achieved their desired/planned number of children and have higher need which often is not met. Conversely, a heightened desire for birth spacing might be projected among younger women, who want to postpone their next pregnancy. Our findings substantiate results from other studies [24–29].

Our study’s findings highlight a significant discrepancy in the unmet need for FP, indicating that the rural women exhibit a greater unmet need for FP compared to their urban counterparts. These findings align with a study conducted in Ethiopia [30], underscoring the existence of discrepancies in family planning unmet needs between urban and rural contexts. Possible explanation for urban/rural differences could be attributed to cultural and behavioral influences along with far off location of facilities that are not equipped with all methods of FP and services interrupted by lack of commodities and problems in the supply chain could bring about this disparity.

Women belonging to the Dalit Ethnicity have slightly higher odds of unmet need for spacing compared to those from the Brahmin/Chettri. Similarly, non-Hindu women have higher odds of unmet need for FP, unmet need for limiting and spacing compared to their Hindu counterparts, and these findings also align with a study conducted in Bihar, India that revealed differences based on ethnicity and religion [31]. Religious restrictions on fertility control could potentially serve as a primary factor for the non-adoption of family planning methods. The interplay of cultural, religious and social factors likely contributed to these observed patterns.

Prior studies have shown that women with advanced education are less likely to have unmet need for spacing, limiting and family planning as evidenced across various global contexts [26, 27, 32, 33]. Contrary to expectations, our study unveils that women with basic and secondary education were more likely to have unmet need for FP compared to those who have no formal education. An alternative explanation for this could be because women with higher educational level have better understanding of the menstruation cycle and unsafe periods, as well as may have more concerns relating to potential side effects of modern contraceptives, which is corroborated by the findings that rhythm and withdrawal methods are more common among women having basic, secondary level or higher education compared to those who had no formal education.

Women hailing from wealthier households tend to display reduced levels of unmet need for family planning, unmet need for spacing and limiting. This finding echoes findings from earlier studies conducted in diverse geographical regions, such as Pakistan [34], Nigeria [35], and Sub-Saharan Africa [36]. A plausible explanation for this pattern is the enhanced accessibility of modern contraceptive methods and the heightened empowerment and autonomy among women from wealthier households as compared from women from poorer households.

Moreover, the study unveiled that woman who had no access to media exhibited a higher likelihood of unmet need for family planning in comparison to those who had access to media. These findings align with findings from prior studies conducted in Mozambique [37, 38] as well as Ethiopia [39–41]. A probable justification for this lies in the potential of media access to counter prevailing misconceptions that impact contraceptive utilization through the facilitation of behavioral transformations.

The study shows a promising opportunity to enhance access to family planning services but calls for specific and targeted actions. A key approach could be to focus on strategy that generates demand, aiming to empower healthcare seeking behaviors, particularly among marginalized women. Policy makers and managers could design programs that provide women with personalized counseling on the full range of contraceptive options which allows them to choose methods that align with their unique situations and aspirations, with flexibility to switch methods as needed. These efforts could extend to both men and women, creating an environment where both partners feel comfortable seeking support and encouraging open conversations about family planning. By building upon the existing policies, Nepal has the potential to drive considerable progress in reducing unmet need for family planning, progressing towards achieving sustainable development goals.

### Strengths and limitations

There are several strengths of this study. Firstly, this study utilized a nationally representative dataset to examine the factors associated with unmet need for family planning among currently married women in Nepal, enhancing the generalizability of its findings across the nation. Secondly, the use of weighted analysis effectively accounted for the complex survey design of the NDHS survey. However, certain limitations warrant consideration. Primarily, the study exclusively focused on the demand side factors and does not consider the supply side factors like the availability of family planning methods or counseling, provider competency, or quality of care. Secondly, potential recall bias may influence the study as women may give socially acceptable responses and may find it difficult to recall past experiences. Lastly, the cross-sectional nature of the data impedes the establishment of causal relationship between dependent and independent variables.

## Conclusion

The study found that many factors shape the unmet need for family planning among currently married women in Nepal. Findings uncovered intriguing patterns that cut across age groups, education levels, wealth differences and belonging to marginalized population. These findings emphasize the necessity of tailored interventions that cater to the unique circumstances of these women. Moreover, strengthening the grassroots-level women development initiatives becomes essential. This involves strengthening the support of local advocates who can pinpoint barriers to accessing services and enhance healthcare availability by spreading essential health information to the targeted population, aiming to accomplish the targets outlined in the Sustainable Development Goals.
